# Initial experience with a virtual atrial fibrillation clinic after pulmonary vein isolation using follow-up with photoplethysmography

**DOI:** 10.1007/s12471-025-01935-6

**Published:** 2025-02-11

**Authors:** Melanie Reijrink-de Boer, Iris Wolsink, Irene Frenaij, Kasper F. Beukema, Berber Brouns, Vincent F. van Dijk, Max Liebregts, Maurits C. E. F. Wijffels, Lucas V. A. Boersma, Jippe C. Balt

**Affiliations:** 1 Department of Cardiology, St. Antonius Hospital, Nieuwegein, The Netherlands; 2 Department of Cardiology, Isala Hospital, Zwolle, The Netherlands; 3 Department of Cardiology, Amsterdam University Medical Centres, Amsterdam, The Netherlands

**Keywords:** Atrial fibrillation, Pulmonary vein isolation, Telemonitoring, Photoplethysmography

## Abstract

**Background:**

To detect recurrent atrial fibrillation (AF) after pulmonary vein isolation (PVI), different methods can be used, ranging from incidental electrocardiograms (ECGs) to rhythm monitoring with implantable loop recorders. We investigated whether telemonitoring (TM) with photoplethysmography (PPG) is feasible for post-PVI follow-up.

**Methods:**

In total, 157 pre-PVI patients were included. Of them, 78 underwent TM at a virtual AF clinic, for which they received a PPG application and were monitored by trained eNurses. The numbers of hospital contacts, hospital visits, ECGs and Holter recordings were assessed. Patient satisfaction and quality of life were analysed. Comparisons were made with a historical control group with a traditional follow-up of outpatient visits, ECGs and Holter recordings (*n* = 79).

**Results:**

Mean ± standard deviation (SD) age was 63 ± 10 years, and 64% were male. AF was paroxysmal in 68% of the patients. Follow-up at 1 year was completed in all patients. In the TM group, the mean ± SD annual number of recordings per patient was 16 ± 29, and AF was detected in 37 patients (47%). The TM group experienced significant decreases in the numbers of unplanned outpatient clinic visits and AF-related hospital admissions, as well as reductions in the numbers of ECGs and Holter recordings performed. Patients reported high satisfaction with this form of TM.

**Conclusion:**

The use of a virtual AF clinic was feasible, and satisfaction was high. Compared with patients with a traditional follow-up, patients on PPG-based TM needed fewer hospital visits and admissions and underwent fewer ECGs and Holter recordings.

**Supplementary Information:**

The online version of this article (10.1007/s12471-025-01935-6) contains supplementary material, which is available to authorized users.

## What’s new?

- This study confirmed the feasibility of a virtual atrial fibrillation (AF) clinic for monitoring patients after pulmonary vein isolation (PVI) using photoplethysmography (PPG).
- This approach reduced the need for frequent healthcare contacts (i.e. fewer planned and unplanned hospital visits, admissions, electrocardiograms and Holter recordings) while providing high-quality patient care.
- Patient satisfaction with the virtual AF clinic was notably high, with PPG-based monitoring deemed to be user-friendly and therapeutically beneficial.
- Patient instructions are crucial for effective monitoring.
- PPG-based heart rhythm monitoring with predefined alerts and the option for patient contact with trained eNurses are recommended for follow-up after PVI.

## Introduction

Atrial fibrillation (AF) is the most common cardiac arrhythmia, and its prevalence is expected to increase over the next decades [1]. This arrhythmia can significantly affect patients’ daily functioning and quality of life (QoL) [2]. Moreover, AF is associated with substantial morbidity and related healthcare consumption [3]. The primary goal of AF treatment is to prevent stroke, alleviate AF-related symptoms and enhance the overall QoL [4].

If initial treatment with anti-arrhythmic drugs fails to restore and sustain sinus rhythm, pulmonary vein isolation (PVI) is now considered to be the indicated therapy for AF [5]. With an expected surge in ablation procedures in the coming years, it is crucial to monitor the utilisation and efficacy of this procedure. Procedural success is typically gauged by the percentage of patients achieving freedom from recurrent AF after PVI. However, this rate varies among studies due to differences in monitoring intensity and follow-up duration [6].

Various methods are employed to detect recurrent AF, ranging from intermittent electrocardiograms (ECGs) to continuous rhythm monitoring with implantable loop recorders (ILRs). However, routine use of ILRs in clinical practice is hindered by cost constraints. Repeated Holter monitoring poses a significant burden on both patients and caregivers, and its efficacy in detecting (recurrent) AF has been shown to be suboptimal [7].

The emergence of photoplethysmography (PPG), utilising the cameras of smartphones and smartwatches, presents a promising, accessible technology for heart rate and rhythm assessment [8, 9]. PPG-based rhythm detection is increasingly integrated into clinical practice and may have a role in post-AF ablation follow-up. AF can be promptly detected when symptoms occur, enabling patients to receive instant feedback on AF presence. Moreover, PPG recordings can be shared with caregivers via digital health platforms. Initial experiences with a virtual AF clinic utilising PPG were reported [10, 11], but no long-term follow-up data after AF ablation have been published.

Despite its potential, it remains uncertain whether post-ablation PPG-based monitoring is feasible [5, 12]. Questions persist regarding its impact on hospital staff workload, the potential reduction in in- and outpatient clinical encounters and patient satisfaction with this form of monitoring.

A virtual post-ablation AF clinic was established, providing remote follow-up with PPG integrated into a digital health platform. The impact of this follow-up method on healthcare utilisation, patient satisfaction and AF-related QoL was investigated. This study focused on the patients enrolled in the first 3 months of the virtual AF clinic who completed 1 year of follow-up, with a comparison with a control group of patients who underwent AF ablation without remote monitoring. Portions of this manuscript were presented at the European Heart Rhythm Association Congress in April 2024 [13].

## Methods

### Study design

This was a retrospective observational study. The St. Antonius Hospital’s medical ethics committee approved the study protocol (W23.069).

### Study population

#### Telemonitoring group

From February through May 2022, 78 patients who underwent PVI were enrolled, and remote follow-up was conducted using PPG. Remote monitoring was overseen by a team of trained eNurses stationed at a telemonitoring centre (TMC). ENurses are nurses trained on specific topics, such as AF. They are able to answer AF-related questions from patients about symptoms, heart rate and heart rhythm and can give medication advise. The eNurses worked online and monitored the measurements from participants remotely but did not perform face-to-face consultations. On-call cardiologists were available to answer questions from eNurses and prescribed medication.

#### Control group

The control group comprised 79 patients who underwent PVI between January and April 2019 as a pre-COVID cohort. Follow-up was performed according to standard care, consisting of a visit to the outpatient department at 3 months, during which an ECG was performed to detect AF or sinus rhythm. Referred patients returned to the referral hospital, while local patients had a 12-month outpatient visit. Holter monitoring was performed at the discretion of the treating cardiologist.

### Study procedures

#### Onboarding telemonitoring group

At the virtual AF clinic, patients received written information on the background and objectives of remote monitoring using TM. They were instructed to download the PPG application, create an account with a two-step verification and perform a test registration, during which they were supported by means of an informative brochure with step-by-step instructions. The brochure also provided information about heart rhythm measurements. Patients were informed they could perform a heart rhythm measurement when it was convenient or if they experienced symptoms. Furthermore, for each measurement, they could indicate whether symptoms were present at that time. The brochure gave tips for performing measurements as well, such as keeping their phone on silent during measurement, ensuring their hands were warm and not talking during the measurement. The application also offered the possibility to contact the healthcare provider if the patient had any questions and read information about heart diseases and medication. Additional support and informed consents were provided by eNurses.

Additionally, patients received questionnaires via email during onboarding and shortly before offboarding, again with the option to contact the eNurses if desired.

#### Photoplethysmography application in telemonitoring group

For AF detection, a smartphone-based PPG application (Happitech, Rotterdam, the Netherlands) was used (see in Figure S1 in Electronic Supplementary Material), employing an algorithm previously described [8]. In short, this algorithm utilises peak detection to measure RR intervals, determining the heart frequency and quality of measurements while providing a rhythm classification (regular (= 0, implying sinus rhythm) or irregular (= 1, implying AF)). Patients can report AF-related symptoms in the application, which is integrated into a digital health platform (Luscii, Utrecht, the Netherlands).

**Fig. 1:**
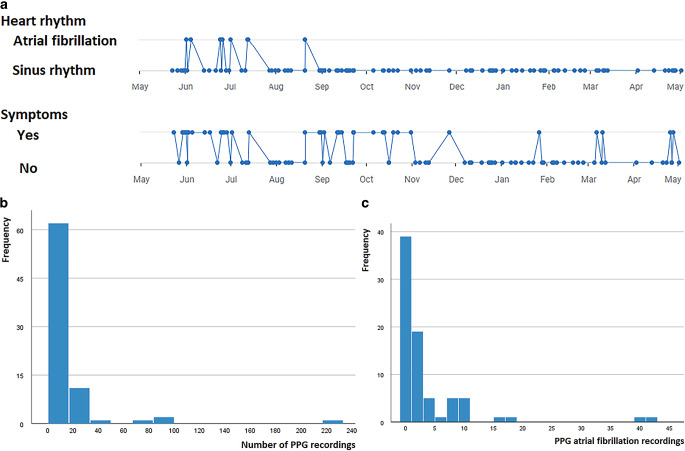
Photoplethysmography (*PPG*). **a** Time plots of rhythm classification and symptoms, as shown in digital health platform Luscii, **b** number of PPG recordings and **c** number of PPG-determined atrial fibrillation recordings

Study participants used the application for heart rhythm measurements if they experienced symptoms. The results of each measurement were displayed and automatically uploaded to a server, accessible from the electronic health record (EHR; Epic Hyperspace, Verona, WI, USA). In the EHR, cardiologists could access patient summaries, which displayed the PVI date, measurements, rhythm classification and symptoms on a time scale (Fig. 1a). Drill-down options allowed access to individual measurements.

#### Offboarding telemonitoring group

The monitoring period ended at 12 months. The numbers of measurements with and without AF were noted, and a report of these findings was documented in the EHR and sent to the referral hospital.

A validated AF patient-specific satisfaction questionnaire, the Atrial Fibrillation Effect on QualiTy-of-Life (AFEQT) questionnaire, was sent to all patients at the start of the programme (baseline) and after 1 year of follow-up [14]. The AFEQT questionnaire includes questions about how AF affects patients’ daily activities, symptoms, treatment satisfaction and overall well-being. In this study, we used only the overall score.

### Data collection and analysis

#### Protocol

TM patients were instructed to perform measurements if symptomatic and were free to perform measurements at any other time. No blanking period was observed. Patients were informed that the TMC would be alerted if their heart frequency was < 30 or > 140 bpm. Individual measurements were not routinely assessed. Patients were instructed to contact the TMC for application-related, PPG-related or AF-related questions or problems. A supervising cardiologist oversaw the TMC during office hours, 5 days a week. The TMC was staffed 7 days a week during office hours.

The online Luscii platform was used for the TM of study participants. The platform was accessible 24/7 by all eNurses and cardiologists, including on-call cardiologists. Vital functions, such as heart rate, heart rhythm and symptoms (in the current version, this was a binary variable, i.e. yes or no), were visible when participants performed PPG measurements. Additionally, participants were able to contact eNurses in the Luscii platform via online chat.

At 3 months, a remote consultation with the cardiologist was scheduled, including a discussion of the PPG measurements. If the patient was free of AF symptoms, anti-arrhythmic drugs were stopped. After this consultation, patients were referred back to the referral hospital for a 12-month follow-up or, for local patients, a 12-month consultation was scheduled in our hospital. These scheduled consultations at 3 and 12 months were part of the planned remote contacts. Patients received a questionnaire containing questions whether follow-up by PPG met their needs (see 2 Dutch-language questionnaires in Electronic Supplementary Material).

Data were collected using the Research Electronic Data Capture system. Custom-built electronic case report forms ensured real-time data validation and integrity checks.

#### Analysis

For the TM group, PPG measurements and contacts with the TMC were documented, as well as the patient satisfaction results. In all patients, both scheduled and unscheduled remote contacts and outpatient contacts, emergency room presentations, cardioversions and AF-related hospital admissions were documented, together with the numbers of ECGs, event recorders and Holter recordings. The number of hospital contacts in our hospital was collected manually by EHR assessment. The hospital contacts in the referral centres were not included in the analysis. This method was similar in both study groups. Follow-up was conducted at 1 year in all patients.

### Statistical analysis

Data from all included participants were analysed, and missing values were not imputed. Data are presented as mean ± standard deviation (SD) for normally distributed continuous variables, median (interquartile range; IQR) for non-normally distributed variables and number (percentage) for categorical variables. As this was an observational study, there was no formal hypothesis or statistical plan, and no power calculation was performed. Continuous data were compared using the unpaired *t*-test or Mann-Whitney U test for two-group comparisons. Categorical data were compared using the χ^2^ test or Fisher’s exact test when a small number of events was observed. Event-free survival is graphically depicted using the Kaplan-Meier method. All tests were two-tailed, and the limit for statistical significance was set at *p* < 0.05. Statistical analysis was performed using SPSS version 28.0.1.0 (IBM, Armonk, NY, USA).

## Results

**Fig. 2:**
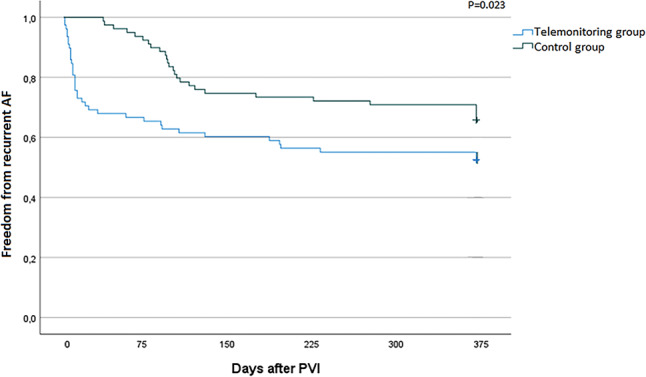
Time to first atrial fibrillation (*AF*) recurrence within first 12 months of pulmonary vein isolation (*PVI*)

In total, 157 patients were enrolled (78 in the TM group and 79 in the control group). Except for the PVI method and beta-blocker use, baseline characteristics did not differ between the 2 groups (Table 1). In both groups, radiofrequency ablation was the leading PVI method. However, in the TM group, cryoablation was also used (21% of cases). Mean ± SD age was 63 ± 10 years, and 63–65% of the patients were male. Documented recurrent AF showed a trend towards lower incidence in the control group compared with the TM group (35% vs 47%; *p* = 0.146). Figure 2 demonstrates that AF was detected earlier in the TM group compared with the control group (*p* = 0.023).

**Table 1:** Baseline characteristics

Variable	Telemonitoring (*n* = 78)	Control (*n* = 79)	*P*-value^a^
Age, years	63 ± 10	63 ± 10	0.847
Men	51 (65)	50 (63)	0.785
Paroxysmal AF	48 (62)	58 (73)	0.113
Primary PVI	72 (92)	73 (92)	0.964
CHA2DS2-VASc score	1.5 (0.8–2.0)	2.0 (1.0–3.0)	0.339
LVEF	54 ± 6.0	54 ± 7.0	0.299
LAVI, ml	32 ± 10	33 ± 6.4	0.538
MR^b^	13 (17)	13 (17)	0.245
Beta-blocker use at baseline	13 (17)	19 (24)	** 0.005**
Beta-blocker use at 1‑year follow-up	13 (17)	19 (24)	** 0.036**
Number of PPG recordings	16 (1–220)		
Number of PPG recordings with AF	3 (1–48)		
*PVI method*			**<** **0.001**
RF	79%	100%	
Cryoablation	21%	0%	
Anti-arrhythmic medication, classes I and III	71%	63%	0.336

Data are mean ± standard deviation. *n* (%), median (interquartile range) or %

^a^ Significant *p*-values are shown in bold

^b^ Percentage of mitral regurgitation (*MR*) grade > 1

*AF* atrial fibrillation, *PVI* pulmonary vein isolation, *LVEF* left ventricular ejection fraction, *LAVI* left atrial volume index, *PPG* photoplethysmography, *RF* radiofrequency ablation

No statistically significant difference was observed in the utilisation of anti-arrhythmic medications at 1‑year follow-up: the prevalence of classes I and III anti-arrhythmic drug use was 23% in the TM group and 26% in the control group (Table 1).

### Photoplethysmography

In the TM group, the mean ± SD annual number of recordings was 16 ± 29 (range: 1–220) (Fig. 1b). In 37 patients (47%), AF was detected. The mean number of AF PPG detections was 3 (range: 1–48). Figure 1c shows the PPG-determined AF recordings. Figure S2 illustrates PPG-detected AF, while Figure S3 shows PPG-detected sinus rhythm (see Electronic Supplementary Material).

Figure 1a is a screenshot of the digital health platform Luscii. It displays the time plots of rhythm classification and symptoms. This figure demonstrates that these time plots do not match 100%, which can be explained by asymptomatic AF episodes. In 208 of the 288 (72%) AF recordings, patients were symptomatic. On the other hand, in 232 of the 952 (24%) recordings, patients did experience symptoms, even when their heart rhythm was regular.

### Healthcare contacts

The mean ± SD number of patient contacts with the virtual AF clinic per year was 0.53 ± 0.66 (range: 0–3). This included scheduled consultations at 3 and 12 months. Of the TM patients, 77% did not contact the virtual AF clinic, 21% had 1 contact, 1% had 2 contacts, and 1% had 3 contacts.

The numbers of planned and unplanned hospital contacts were both higher during the first year of follow-up after PVI in the control group than the TM group, in contrast to the number of planned remote contacts. The number of unplanned remote contacts did not differ significantly between the groups. As shown in Table 2, the median (IQR) number of planned hospital contacts per patient was 0 (0–0) in the TM group and 1 (1–1) in the control group (*p* = 0.001), whereas the number of patients with unplanned hospital contacts was 4 (5%) and 20 (25%), respectively (*p* < 0.001). The mean ± SD number of planned remote contacts per patient was 1 ± 0.62 in the TM group and 0.23 ± 0.53 in the control group (*p* < 0.001), and the mean ± SD number of unplanned remote contacts was 0.78 ± 1.16 and 0.70 ± 1.3, respectively (*p* = 0.332).

**Table 2:** Healthcare contacts and questionnaires

Variable	Telemonitoring (*n* = 78)	Control (*n* = 79)	*P*-value^a^
Total number of planned and unplanned remote and outpatient contacts	2 (1–3)	2 (1–3)	0.172
Number of planned remote contacts	1 (1–1)	0 (0–0)	**<** **0.001**
Number of planned outpatient clinic visits	0 (0–0)	1 (1–1)	** 0.001**
Patients with ≥ 1 unplanned remote contacts	33 (42)	26 (32)	0.178
Patients with ≥ 1 unplanned outpatient clinic visits	4 (5)	20 (25)	**<** **0.001**
Patients with ≥ 1 virtual AF clinic contacts	36 (46)		
Patients with ≥ 1 detected recurrent AF episodes	37 (47)	28 (35)	0.127
Patients with ≥ 1 electrical cardioversions	6 (8)	7 (9)	0.791
Patients with ≥ 1 emergency (cardiac) care unit visits for AF	8 (10)	7 (9)	0.766
Patients with ≥ 1 AF-related hospital admissions	1 (1)	13 (16)	** 0.001**
Patients with ≥ 1 ECGs	44 (56)	78 (99)	**<** **0.001**
Patients with ≥ 1 event recorders	0 (0)	1 (1)	0.317
Patients with ≥ 1 Holter recordings	3 (4)	13 (16)	** 0.012**
AFEQT score at baseline	59 ± 20^b^	52 ± 21^c^	0.063
AFEQT score at 1‑year follow-up	79 ± 23^d^	76 ± 21^e^	0.966

Data are median (interquartile range), *n* (%) or mean ± standard deviation

^a^ Significant *p*-values are shown in bold

^b^ *n* = 58

^c^ *n* = 69

^d^ *n* = 13

^e^ *n* = 57

*AF* atrial fibrillation, *AFEQT* Atrial Fibrillation Effect on QualiTy-of-Life questionnaire, *ECG* electrocardiogram

The primary reasons why patients contacted the TMC were symptoms such as palpitations, dyspnoea, fatigue or thoracic or groin pain after an ablation, with the request to see or speak to a healthcare provider. Secondary reasons were questions about the Luscii application or onboarding or technical questions about PPG measurements.

The mean ± SD number of ECGs was higher in the control group than the TM group (3.27 ± 2.51 vs 1.18 ± 1.8; *p* < 0.001), as was the mean ± SD number of Holter recordings (0.18 ± 0.42 vs 0.04 ± 0.19; *p* = 0.004).

### Patient satisfaction

**Fig. 3:**
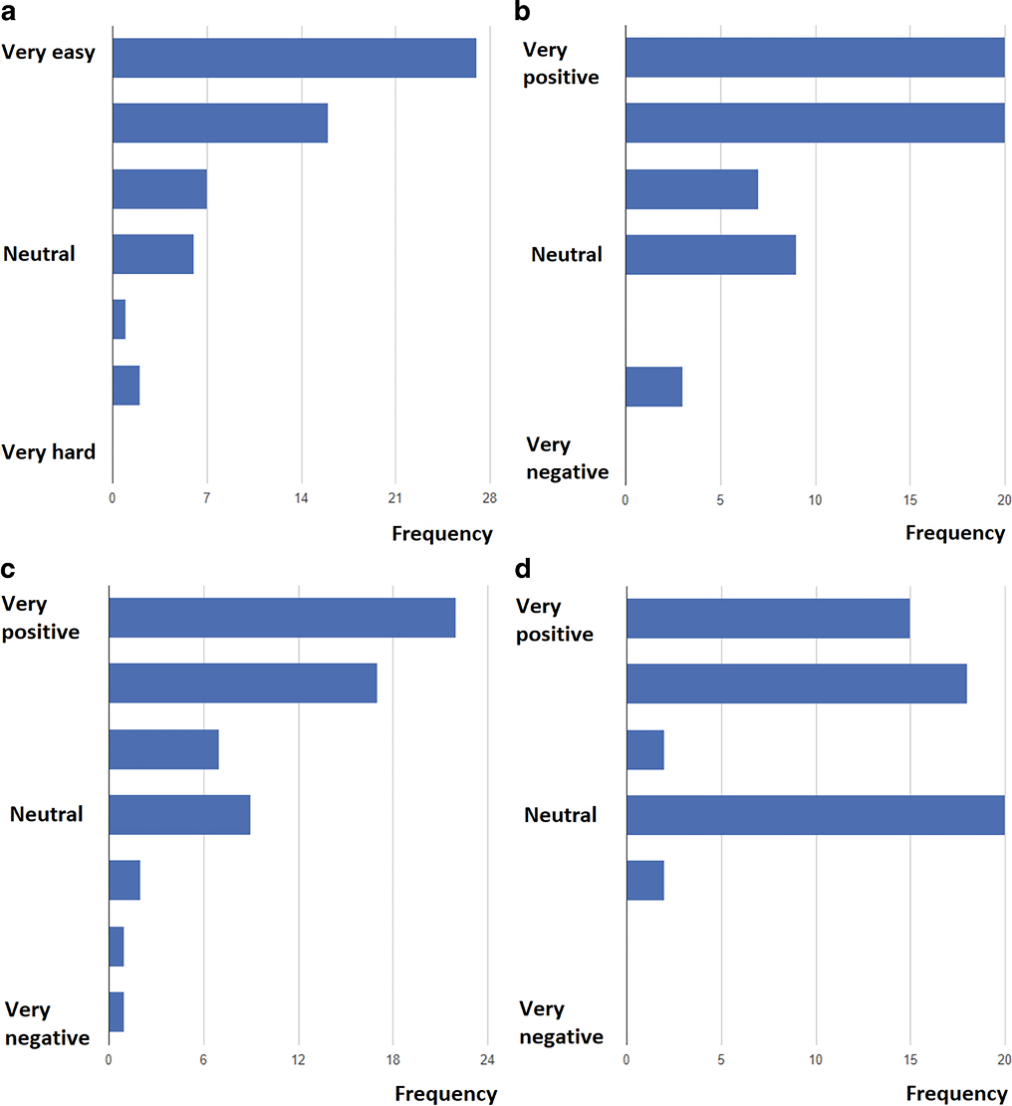
Results of questionnaire on patient satisfaction with use of photoplethysmography application, displayed on 7‑point scale, with regard to **a** ease of use (question: ‘How do you experience using the application to make a successful registration?’), **b** heart rhythm control (question: ‘How do you experience the sense of control in self-monitoring your heart rhythm?’), **c** fit with patient needs (question: ‘Does this method of rhythm monitoring suit your situation/needs?’) and **d** beneficial effect on atrial fibrillation treatment (question: ‘Is the use of the application an added value to your AF treatment?’)

In the TM group, 57 patients (73%) completed the questionnaire on satisfaction with TM. As indicated in Fig. 3, patients expressed positive sentiments about the use of the PPG application and the virtual AF clinic. They thought the application was very easy to use, were positive about heart rhythm control and believed the follow-up fitted their needs and benefitted their AF treatment. Some questions were answered mostly ‘neutral’.

AFEQT questionnaires were completed by 58 of the patients (74%) in the TM group and 69 (87%) in the control group at baseline and by 13 (17%) and 57 (72%), respectively, after 1 year of follow-up. Quality of life (measured with AFEQT) did not differ between the groups.

## Discussion

In this study, a virtual AF clinic for post-PVI patients was evaluated, aimed at providing high-quality monitoring and patient care without an overwhelming number of healthcare contacts. The impact of the virtual AF clinic on healthcare outcomes were presented herein. This analysis focused on the initial patients completing 1‑year follow-up and demonstrated the feasibility of heart rhythm monitoring with PPG in post-PVI AF patients. Engagement with a virtual AF clinic did not lead to an excessive number of healthcare contacts. In fact, there were fewer planned and unplanned hospital visits, hospital admissions, ECGs and Holter recordings in the TM group compared with the control group, consistent with prior research [15]. For instance, the virtual AF clinic TeleCheck-AF study by Gawałko et al. showed significant reductions in the numbers of ECGs, Holter recordings and echocardiograms [15].

### Patient adherence

Patients were instructed to perform PPG measurements if symptomatic, without specifying a required number of recordings. Consequently, a mean annual number of 16 PPG registrations per patient was recorded, with a wide range (1–220). Apparently, patients performed few measurements when instructed to record heart rhythm only when symptomatic. In contrast, Van der Velden et al. instructed patients to perform 3 daily measurements for 4 weeks, resulting in daily measurements in 74% of the study population [16]. In the current study, recurrent AF, as anticipated, was detected earlier in the TM group. The frequency of AF detection increased in tandem with the frequency of heart rhythm measurements [12].

### Photoplethysmography telemonitoring at a telemonitoring centre

PPG was chosen for AF detection due to its cost-effectiveness and, in its present form, a seamless integration into a digital health platform. The sensitivity and specificity of PPG for AF detection are promising (94.2 and 95.8%, respectively) [17]. The success of PPG monitoring in AF ablation has been shown before. In an 8‑week follow-up study, Gruwez et al. demonstrated a sensitivity of 98.3%, a specificity of 99.9%, a positive predictive value of 99.6% and a negative predictive value of 99.6% for PPG-based AF detection after AF ablation [18]. As a limitation, they noted an underestimation of higher heart rates.

In our study, the digital health platform provided safety features such as alerts for bradycardia and tachycardia. Keeping the underestimation of higher heart rates in mind, the upper limit for heart rhythms was set at 140 bpm. The accessibility of PPG data in the EHR facilitates cardiologists’ review, which is crucial for embracing this novel AF monitoring approach. Patients were able to contact the TMC via the application or by phone. Contrary to expectations, patient contact with the TMC was limited, indicating clear patient instructions were pivotal. Although cardiologists supervised eNurses and backup was readily available, direct contact between eNurses and referring cardiologists was infrequent.

### Patient satisfaction

Patient satisfaction with the virtual AF clinic was notably high. PPG-based heart rhythm monitoring was deemed user-friendly, and its therapeutic benefits for AF treatment were highly regarded. The possibility of recording AF episodes- and the option of sharing these recordings in real time with their caregivers—gives patients more control over their disease. Paroxysmal episodes can be appreciated and managed as such (with or without the help of extra anti-arrhythmic drug use), and persistent episodes are easier to share with the hospital. ENurses can advise patients on restarting or increasing their anti-arrhythmic drugs or arrange a cardioversion, without a visit to the cardiac emergency care unit, as is often the case without TM. In short, patients feel empowered regarding the management of AF.

Previous studies on AF clinics have reported similar benefits for patients [19, 20]. Some patients ‘neutral’ to questions on contact with the TMC or application use. These results may be interpreted as ‘no opinion’ due to the absence of contact with the eNurses or the absence of AF. However, patients with TMC contact and/or AF may also have a neutral opinion. Remote care was administrated by a team of trained eNurses at the TMC, a model akin to monitoring patients with pacemakers or implantable cardioverter-defibrillators [21, 22] or as described for PPG-based AF care during the COVID-19 pandemic [10]. In the latter case, patients with diagnosed or suspected AF were followed for a few weeks. In our post-PVI programme, patients were monitored for a year following PVI, aiming to enhance care quality and rhythm follow-up while minimising the numbers of hospital visits and admissions.

### Study limitations

Although a historical control group was used for comparison, randomisation was not performed. Hence, our results can be hypothesis-generating only. Additionally, there was potential underreporting of recurrences in the TM group since patients were requested to perform measurements if symptomatic or if they wanted to. Requesting periodic (weekly) measurements could have yielded more data, also on asymptomatic AF recurrences. Furthermore, potential bias may have been introduced by a difference in the use of ablation techniques between the TM and control groups.

Inherently, PPG provides limited information compared with ECG. The heart rhythms were classified as regular or irregular, the latter implying AF. PPG is adequate for detection of AF, but misclassification of premature complexes as irregular or atrial flutter as regular may have occurred. As (atypical or left atrial) flutter may arise after PVI, a high index of suspicion is required for detecting atrial flutter. There were 3 patients with atrial flutter after PVI, all of whom were identified by the presence of the combination of a regular rhythm > 100 bpm. Further studies should assess PPG sensitivity and specificity post-PVI, e.g. by correlating PPG findings with implantable device data. Next to that, symptom reporting by patients was binary. For further research, a determination of the type of symptoms may be insightful. In this analysis of patients included in the initial phase of the TM programme, a cost-effectiveness assessment was not performed yet. Clear patient instructions regarding periodic PPG recordings are essential for optimal AF detection and monitoring.

## Conclusion

The use of a virtual AF clinic for post-PVI patients was feasible and resulted in precise AF detection, high patient satisfaction and fewer hospital visits. Based on this study, PPG-based heart rhythm monitoring with predefined alerts and the possibility for patients to contact trained e‑Nurses are recommended as a follow-up method after PVI.

## Supplementary Information

Figure S1 PPG detection

Figure S2 PPG-detected irregular rhythm

Figure S3 PPG-detected regular rhythm

Questionnaire 1

Questionnaire 2

## References

[1] Lippi G, Sanchis-Gomar F, Cervellin G. Global epidemiology of atrial fibrillation: an increasing epidemic and public health challenge. Int J Stroke. 2021;16:217–21. 31955707 10.1177/1747493019897870

[2] Thrall G, Lane D, Carroll D, et al. Quality of life in patients with atrial fibrillation: a systematic review. Am J Med. 2006;119:e1–e19. 10.1016/j.amjmed.2005.10.057 16651058

[3] Andrade JG, Deyell MW, Macle L, et al. Healthcare utilization and quality of life for atrial fibrillation burden: the CIRCA-DOSE study. Eur Heart J. 2023;44:765–76. 36459112 10.1093/eurheartj/ehac692

[4] Wolfes J, Ellermann C, Frommeyer G, et al. Evidence-based treatment of atrial fibrillation around the globe: comparison of the latest ESC, AHA/ACC/HRS, and CCS guidelines on the management of atrial fibrillation. Rev Cardiovasc Med. 2022;23:56. 35229547 10.31083/j.rcm2302056

[5] Hindricks G, Potpara T, Dagres N, et al. ESC scientific document group. 2020 ESC guidelines for the diagnosis and management of atrial fibrillation developed in collaboration with the European association for Cardio-thoracic surgery (EACTS): the task force for the diagnosis and management of atrial fibrillation of the European society of cardiology (ESC) developed with the special contribution of the European heart rhythm association (EHRA) of the ESC. Eur Heart J. 2021;42:373–498. 32860505 10.1093/eurheartj/ehaa612

[6] Aguilar M, et al. Influence of monitoring strategy on assessment of ablation success and postablation atrial fibrillation burden assessment: implications for practice and clinical trial design. Circulation. 2022;145:21–30. 34816727 10.1161/CIRCULATIONAHA.121.056109

[7] Hanke T, Charitos EI, Stierle U, et al. Twenty-four-hour holter monitor follow-up does not provide accurate heart rhythm status after surgical atrial fibrillation ablation therapy: up to 12 months experience with a novel permanently implantable heart rhythm monitor device. Circulation 2009;120(Suppl. 11):S177–S84. 10.1161/CIRCULATIONAHA.108.838474 19752365

[8] Mol D, Riezebos RK, Marquering HA, et al. Performance of an automated photoplethysmography-based artificial intelligence algorithm to detect atrial fibrillation. Cardiovasc Digit Health J. 2020;1:107–10. 35265881 10.1016/j.cvdhj.2020.08.004 PMC8890349

[9] Guo Y, Wang H, Zhang H, et al. Mobile photoplethysmographic technology to detect atrial fibrillation. J Am Coll Cardiol. 2019;74:2365–75. 31487545 10.1016/j.jacc.2019.08.019

[10] Van der Velden RMJ, Verhaert DVM, Hermans ANL, et al. The photoplethysmography dictionary: practical guidance on signal interpretation and clinical scenarios from telecheck-AF. Eur Heart J Digit Health. 2021;2:363–73. 36713592 10.1093/ehjdh/ztab050 PMC9707923

[11] Manninger M, Hermans ANL, Caracioni AA, et al. Photoplethysmography-documented atrial fibrillation in the first week after catheter ablation is associated with lower success rates. Front Cardiovasc Med. 2023;10:1199630. 37424905 10.3389/fcvm.2023.1199630 PMC10324576

[12] Svennberg E, Tjong F, Goette A, et al. How to use digital devices to detect and manage arrhythmias: an EHRA practical guide. Europace. 2022;24:979–1005. 35368065 10.1093/europace/euac038 PMC11636571

[13] Reijrink-De M, Boer M, Wolsink I, Frenaij I, et al. Initial experience with a virtual atrial fibrillation clinic after pulmonary vein isolation. Europace. 2024;26:euae102.599–1.

[14] Spertus J, Dorian P, Bubien R, et al. Development and validation of the atrial fibrillation effect on qualiTy-of-life (AFEQT) questionnaire in patients with atrial fibrillation. Circ Arrhythm Electrophysiol. 2011;4:15–25. 21160035 10.1161/CIRCEP.110.958033

[15] Gawałko M, et al. Changes in healthcare utilisation during implementation of remote atrial fibrillation management: telecheck-AF project. Neth Heart J. 2024;32:130–9. 38214880 10.1007/s12471-023-01836-6 PMC10884376

[16] Van der Velden RMJ, Pluymaekers NAHA, Dudink EAMP, et al. Mobile health adherence for the detection of recurrent recent-onset atrial fibrillation. Heart. 2022;109:26–33. 36322782 10.1136/heartjnl-2022-321346

[17] O’Sullivan JW, Grigg S, Crawford W, et al. Accuracy of smartphone camera applications for detecting atrial fibrillation: a systematic review and meta-analysis. JAMA Netw Open. 2020;3:e202064. 32242908 10.1001/jamanetworkopen.2020.2064 PMC7125433

[18] Gruwez H, et al. Real-world validation of smartphone-based photoplethysmography for rate and rhythm monitoring in atrial fibrillation. Europace. 2024;26:euae65. 38630867 10.1093/europace/euae065 PMC11023210

[19] Pluymaekers NAHA, Hermans ANL, van der Velden RMJ, et al. Implementation of an on-demand app-based heart rate and rhythm monitoring infrastructure for the management of atrial fibrillation through teleconsultation: TeleCheck-AF. Europace. 2021;23:345–52. 32887994 10.1093/europace/euaa201 PMC7499572

[20] Gawałko M, Duncker D, Manninger M, et al. The european telecheck-AF project on remote app-based management of atrial fibrillation during theCOVID-19pandemic: centre and patient experiences. Europace. 2021;23:1003–15. 33822029 10.1093/europace/euab050 PMC8083545

[21] Mabo P, Victor F, Bazin P, et al. A randomized trial of long-term remote monitoring of pacemaker recipients (the COMPAS trial). Eur Heart J. 2012;33:1105–11. 22127418 10.1093/eurheartj/ehr419 PMC3341630

[22] De Simone A, Leoni L, Luzi M, et al. Remote monitoring improves outcome after ICD implantation: the clinical efficacy in the management of heart failure (EFFECT) study. Europace. 2015;17:1267–75. 25842271 10.1093/europace/euu318

