# Supplementary figures and images for: Initial experience with a virtual atrial fibrillation clinic after pulmonary vein isolation using follow-up with photoplethysmography
Source: Neth Heart J. 2025 Feb 11;33(3):85–92. doi: 10.1007/s12471-025-01935-6 (PMC11845631; doi:10.1007/s12471-025-01935-6)

**Figure S1** PPG detection


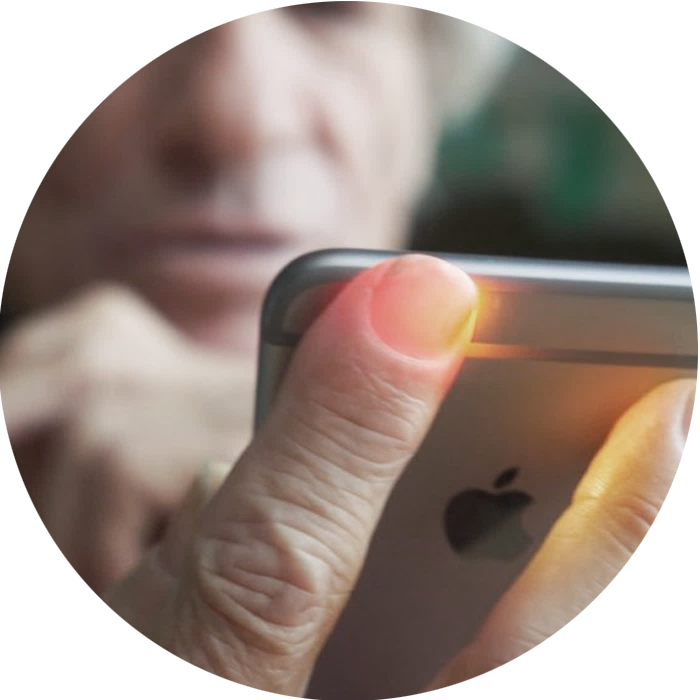

Supplement: Supplementary file 1 — Figure S1 PPG detection [file 12471_2025_1935_MOESM1_ESM.docx]

**Figure S2** PPG-detected irregular rhythm


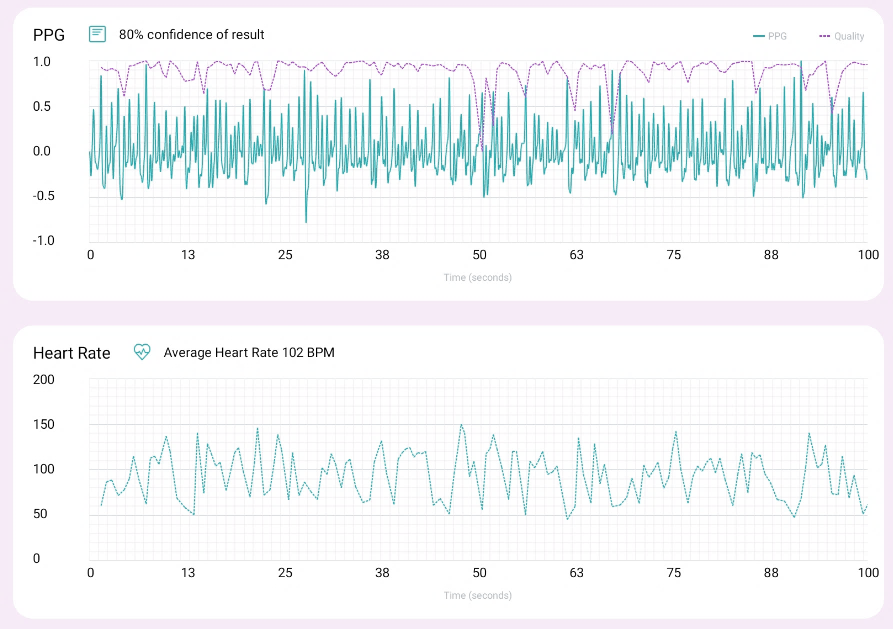

Supplement: Supplementary file 2 — Figure S2 PPG-detected irregular rhythm [file 12471_2025_1935_MOESM2_ESM.docx]

**Figure S3** PPG-detected regular rhythm


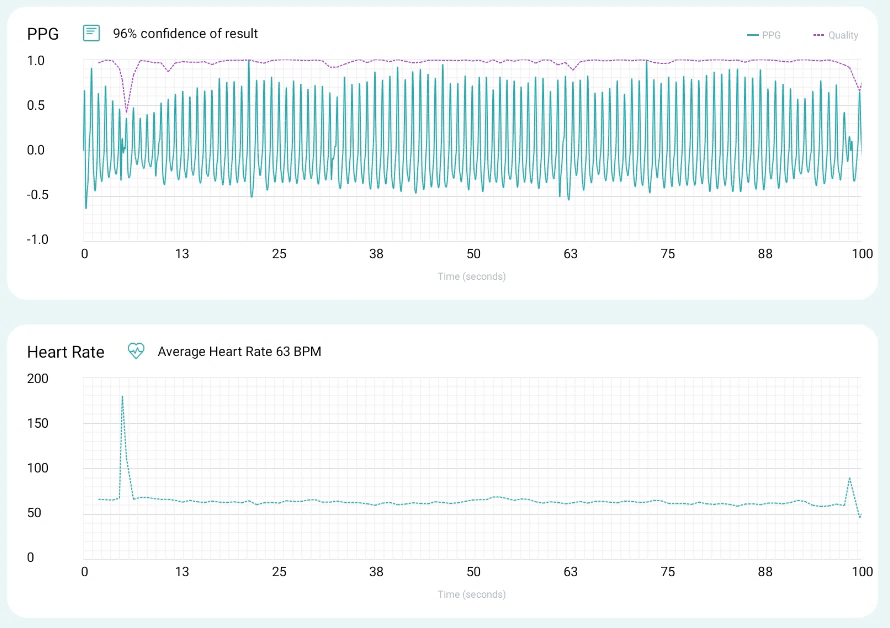

Supplement: Supplementary file 3 — Figure S3 PPG-detected regular rhythm [file 12471_2025_1935_MOESM3_ESM.docx]
