# Supplementary material for: Initial experience with a virtual atrial fibrillation clinic after pulmonary vein isolation using follow-up with photoplethysmography
Source: Neth Heart J. 2025 Feb 11;33(3):85–92. doi: 10.1007/s12471-025-01935-6 (PMC11845631; doi:10.1007/s12471-025-01935-6)
Supplement: Supplementary file 4 — Questionnaire 1 [file 12471_2025_1935_MOESM4_ESM.pdf]

Vragenlijst 2/3: Thuismeten gebruiksgemak en ervaring vragenlijst

Deel 1: De volgende vragen gaan over het gebruik en hanteren van de Thuismeten applicatie.

Geef aan hoe u het gebruik en hanteren ervaart in de volgende situaties:
(1) Heel erg eenvoudig (7) Heel erg moeilijk

	Heel erg Eenvoudig (1)	(2)	(3)	Neutraal (4)	(5)	(6)	Heel erg Moeilijk (7)
1. Het hanteren van de Thuismeten applicatie om een succesvolle registratie te maken?	○	○	○	○	○	○	○
2. Het regelmatig maken van een registratie?	○	○	○	○	○	○	○
3. Het maken van een registratie bij klachten?	○	○	○	○	○	○	○
4. Het contact met het thuismonitoringcentrum bij klachten?	○	○	○	○	○	○	○

Heeft u technische problemen gehad met het registreren van uw hartritme waarvoor u contact heeft gezocht met het thuismonitoringcentrum?

○ ja
○ nee

Hoe vaak heeft u hierover contact gezocht?

____
(Aantal in getal)

Optioneel:
Ruimte voor opmerkingen / feedback over het hanteren en gebruiken van de Thuismeten applicatie.

____

Gebruiksgemak score

____

Deel 2: De volgende vragen gaan over de gebruikservaring van de Thuismeten applicatie bij het monitoren van uw hartritme.
Beoordeel uw ervaring met het gebruik van de Thuismeten applicatie in de volgende situaties: (1) Heel erg positief (7) Heel erg negatief

	Heel erg Positief (1)	(2)	(3)	Neutraal (4)	(5)	(6)	Heel erg Negatief (7)
1. Het zelf monitoren van uw hartritme?	○	○	○	○	○	○	○
2. De invloed op het doen van dagelijkse activiteiten?	○	○	○	○	○	○	○
3. Het gevoel van controle op het monitoren van uw hartritme?	○	○	○	○	○	○	○
4. De dagelijkse bewustwording van en aandacht voor uw hartritme?	○	○	○	○	○	○	○
5. Past deze manier van ritme monitoring bij uw situatie/wensen?	○	○	○	○	○	○	○

Optioneel:
Ruimte voor opmerkingen / feedback naar aanleiding van de gebruikservaring van de Thuismeten applicatie. ______________________________

Ervaring score ______________________

Deel 3: De volgende vragen gaan over het effect van het gebruik van de Thuismeten applicatie op uw behandeling.
Geef aan in welke mate gebruik van de Thuismeten applicatie invloed heeft op de volgende aspecten van uw behandeling:
(1) heel erg positief (7) heel erg negatief

	Positieve invloed/ effect (1)	(2)	(3)	Neutraal (4)	(5)	(6)	Negatieve invloed/ effect (7)
1. Het aantal benodigde zorgcontacten?	○	○	○	○	○	○	○
2. De tijd tot het vastellen van de aard van de klachten / een diagnose?	○	○	○	○	○	○	○
3. De tijd tot het starten of aanpassen van uw behandeling?	○	○	○	○	○	○	○
4. De invloed op medicatiegebruik en het monitoren daarvan?	○	○	○	○	○	○	○
5. De toegevoegde waarde aan uw behandeling	○	○	○	○	○	○	○

Optioneel:
Ruimte voor opmerkingen / feedback over de invloed op uw behandeling door middel van de Thuismeten applicatie. ________________________________

Impact score ____________________________

Deel 4: de laatste vragen gaan over de Thuismeten applicatie in het geheel

1. Zou u deze vorm van monitoring willen doorgebruiken?

◯ Ja
◯ Nee
◯ Geen mening

2. Een totaal beoordeling voor deze manier van monitoren van uw hartritme

Goed Gemiddeld Slecht

(Place a mark on the scale above)

Ruimte voor opmerkingen / feedback

____

Dank u voor het invullen van de vragenlijst, door op 'Submit' te klikken verstuurt u de vragenlijst en wordt u doorgelinkt naar de laatste vragenlijst over zorgcontacten en medicatiegebruik.
