# Supplementary material for: Initial experience with a virtual atrial fibrillation clinic after pulmonary vein isolation using follow-up with photoplethysmography
Source: Neth Heart J. 2025 Feb 11;33(3):85–92. doi: 10.1007/s12471-025-01935-6 (PMC11845631; doi:10.1007/s12471-025-01935-6)
Supplement: Supplementary file 5 — Questionnaire 2 [file 12471_2025_1935_MOESM5_ESM.pdf]

Vragenlijst 3/3: Klachten, zorgcontacten en medicatie

Deel 1: Zorgcontacten in verband met klachten van boezemfibrilleren (extra of ongepland)

Heeft u zelf het gevoel dat het boezemfibrilleren nog/weer aanwezig is? (ongeacht of dit is vastgesteld met een registratie)

- ◯ Ja
- ◯ Nee
- ◯ Weet ik niet / ik twijfel

Hebt u contact gezocht met het Thuismonitoring centrum sinds de ablatie?

- ☐ Nee
- ☐ Ja, eenmaal
- ☐ Ja, vaker dan eens

Heeft het Thuismonitoringcentrum u tijdig geholpen?

- ◯ Ja
- ◯ Nee

Op welk terrein heeft contact met het Thuismonitoringcentrum geholpen?

- ☐ Ziekenhuisbezoek
- ☐ Medicatie
- ☐ Klachten
- ☐ Geruststelling

Contact met het Thuismonitoring centrum heeft er (overwegend) voor gezorgd dat (meerdere antwoorden mogelijk):

- ☐ Een eerste hart hulp bezoek of polibezoek werd ingepland
- ☐ Een ziekenhuis bezoek is voorkomen
- ☐ Medicatie is gewijzigd
- ☐ Medicatie niet is gewijzigd
- ☐ Ik gerustgesteld ben
- ☐ Anders, namelijk...

Contact met het Thuismonitoringcentrum heeft geleid tot...

____

Op een schaal van 0-10, in hoeverre heeft de mogelijkheid om contact op te nemen met het Thuismonitoringcentrum meerwaarde voor u?

- ◯ 0
- ◯ 1
- ◯ 2
- ◯ 3
- ◯ 4
- ◯ 5
- ◯ 6
- ◯ 7
- ◯ 8
- ◯ 9
- ◯ 10

Op een schaal van 0-10, hoe tevreden bent u met het Thuismonitoringcentrum?

- ◯ 0
- ◯ 1
- ◯ 2
- ◯ 3
- ◯ 4
- ◯ 5
- ◯ 6
- ◯ 7
- ◯ 8
- ◯ 9
- ◯ 10

Waar / bij wie komt u op dit moment op controle voor uw boezemfibrilleren? (meerdere antwoorden mogelijk)

- ☐ St. Antonius Ziekenhuis
- ☐ Verwijzend arts
- ☐ Huisarts
- ☐ Nergens
- ☐ Anders namelijk ...

Waar wordt u gecontroleerd?

____

Heeft u een zorgverlener extra of ongepland bezocht in verband met klachten van boezemfibrilleren?

- ◯ Ja
- ◯ Nee

Wie heeft u extra of ongepland bezocht in verband met klachten van boezemfibrilleren? (meerdere antwoorden mogelijk)

- ☐ Huisarts
- ☐ Huisartsenpost
- ☐ Cardioloog (polikliniek)
- ☐ Eerste hart hulp / Hartbewaking
- ☐ Algemeen spoedeisende hulp (SEH)
- ☐ Nee
- ☐ Anders namelijk...

Welke andere zorgverlener heeft u bezocht?

____

Hoeveel keer heeft u de huisarts bezocht in verband met klachten van boezemfibrilleren?

____
(Aantal in getal)

Hoeveel keer heeft u de huisartsenpost bezocht in verband met klachten van boezemfibrilleren?

____
(Aantal in getal)

Hoeveel keer heeft u de cardioloog (polikliniek) bezocht in verband met klachten van boezemfibrilleren?

____
(Aantal in getal)

Hoeveel keer heeft u de Eerste hart hulp / Hartbewaking / Spoed eisende hulp bezocht in verband met klachten van boezemfibrilleren? (aantal)

____
(Aantal in getal)

Hoeveel keer heeft u de spoed eisende hulp (SEH)bezocht in verband met klachten van boezemfibrilleren?

____
(Aantal in getal)

Hoeveel keer heeft u de "[klamed_bezoek_ander]" bezocht in verband met klachten van boezemfibrilleren?

____
(Aantal in getal)

Deel 2: Aanvullende diagnostische onderzoeken naar het hartritme
Het gaat om de periode ná de laatste vragenlijst 4 maanden geleden

Heeft u een onderzoek ondergaan om het hartritme te registreren, anders dan met Luscii?
◯ Ja
◯ Nee

Welk(e) extra onderzoek(en) naar uw hartritme zijn verricht?

- ☐ ECG (hartfilmpje) bij gepland bezoek
- ☐ ECG (hartfilmpje) bij ongepland bezoek (huisartsenpost / SEH / harthulp)
- ☐ Holter (24 uur of langer thuismonitoring)
- ☐ Event recorder (registratie bij klachten)
- ☐ Geimplanteerd device (interne loop recorder, pacemaker, ICD)
- ☐ Anders namelijk...

Is met behulp van het ECG boezemfibrilleren aangetoond?
◯ Ja
◯ Nee

Is met behulp van het holteronderzoek boezemfibrilleren aangetoond?
◯ Ja
◯ Nee

Is met behulp van de event recorder boezemfibrilleren aangetoond?
◯ Ja
◯ Nee

Is met behulp van het geimplanteerde device boezemfibrilleren aangetoond?
◯ Ja
◯ Nee

Welk ander registratiemiddel is er gebruikt?
____

Is met de/het "[klamed_extradiag_ander]" boezemfibrilleren aangetoond?
◯ Ja
◯ Nee

Deel 3: Medicatiegebruik en medicatiewijzigingen
Het gaat om dit moment en de wijzigingen ná de laatste vragenlijst 4 maanden geleden

Welke bloed verdunnende medicatie gebruikt u op dit moment?

◯ Geen ◯ Acenocoumarol (Sintrom) ◯ Fenprocoumon (Marcoumar) ◯ Rivaroxaban (Xarelto)
◯ Apixaban (Eliquis) ◯ Dabigatran (Pradaxa) ◯ Edoxaban (Lixiana) ◯ Anders namelijk...

Andere bloedverdunner(s) namelijk

____

Zijn uw bloedverdunnende medicijnen veranderd in de afgelopen 4 maanden? (gestart/gestopt of dosis wijzigingen)

◯ Ja
◯ Nee

Wat is er gewijzigd aan uw bloedverdunners?
(middel - wijziging - datum)

____

Welke medicijnen gebruikt u voor het hartritme / tegen hartritme stoornissen op dit moment?
(Zo nodig medicatie telt niet mee indien u dit minder dan 2x per week gebruik)

☐ Geen ☐ Flecainide (Tambocor) ☐ Sotalol ☐ Amiodaron (Cordarone) ☐ Verapamil (Isoptin)
☐ Diltiazem (Tildiem) ☐ Disopyramide (Ritmoforine) ☐ Propafenon (Rytmonorm) ☐ Metoprolol, Atenolol, Propranolol, Bisoprolol, Carvedilol of Nebivolol ("Bèta-blokkers") ☐ Digoxine (Lanoxin) ☐ Anders namelijk ...

Andere hartritme medicijn(en) namelijk

____

Zijn uw hartritme medicijnen veranderd in de afgelopen 4 maanden? (gestart/gestopt of dosis wijzigingen)

◯ Ja
◯ Nee

Wat is er gewijzigd aan uw hartritme medicijnen?
(middel - wijziging - datum)

____

Deel 4: Aanvullende behandelingen in het kader van hartritme stoornissen
Het gaat om de periode ná de laatste vragenlijst 4 maanden geleden

Heeft u een (electrische) cardioversies ondergaan?	◯ Ja<br>◯ Nee
Hoe vaak heeft u een (electrische) cardioversie ondergaan (ECV)?	◯ 1 ◯ 2 ◯ 3 ◯ 4<br>◯ 5 ◯ 6 ◯ 7 ◯ 8<br>◯ 9 ◯ 10
Datum eerste cardioversie	__________________________
Was de eerste cardioversie succesvol?	◯ Ja<br>◯ Nee
Datum tweede cardioversie	__________________________
Was de tweede cardioversie succesvol?	◯ Ja<br>◯ Nee
Datum derde cardioversie	__________________________
Was de derde cardioversie succesvol?	◯ Ja<br>◯ Nee
Datum vierde cardioversie	__________________________
Was de vierde cardioversie succesvol?	◯ Ja<br>◯ Nee
Datum vijfde cardioversie	__________________________
Was de vijfde cardioversie succesvol?	◯ Ja<br>◯ Nee
Datum zesde cardioversie	__________________________
Was de zesde cardioversie succesvol?	◯ Ja<br>◯ Nee
Datum zevende cardioversie	__________________________
Was de zevende cardioversie succesvol?	◯ Ja<br>◯ Nee
Datum achtste cardioversie	__________________________

Was de achtste cardioversie succesvol?	◯ Ja<br>◯ Nee
Datum negende cardioversie	______________________________
Was de negende cardioversie succesvol?	◯ Ja<br>◯ Nee
Datum tiende cardioversie	______________________________
Was de tiende cardioversie succesvol?	◯ Ja<br>◯ Nee
Heeft u een aanvullende (tweede) ablatie behandeling ondergaan in verband met hartritmestoornissen? (bijvoorbeeld een katheterablatie of ablatie chirurgie)	◯ Ja<br>◯ Nee
Wat voor herbehandeling heeft u ondergaan?<br><br>◯ Katheterablatie via de lies<br>◯ Chirurgische ablatie<br>◯ Anders namelijk...	
Wat voor type herbehandeling heeft u ondergaan?	______________________________
Wanneer heeft de herbehandeling plaatsgevonden	______________________________
Waar heeft de behandeling plaatsgevonden?<br><br>◯ St. Antonius ziekenhuis<br>◯ Anders namelijk...	
In welk ander ziekenhuis heeft de herbehandeling plaatsgevonden?	______________________________
Heeft u een 2de herbehandeling (derde ablatie) ondergaan in verband met hartritmestoornissen?	◯ Ja<br>◯ Nee
Wat voor 2de herbehandeling heeft u ondergaan?<br><br>◯ Katheterablatie via de lies<br>◯ Chirurgische ablatie<br>◯ Anders namelijk...	
Wat voor type herbehandeling heeft u ondergaan?	______________________________
Wanneer heeft de 2de herbehandeling plaatsgevonden	______________________________

Waar heeft de 2de herbehandeling plaatsgevonden?

◯ St. Antonius ziekenhuis
◯ Anders namelijk...

In welk ander ziekenhuis heeft de 2de herbehandeling plaatsgevonden? __________________________________

Deel 5: Ziekenhuisopnames (cardioversies en (her)ablatiebehandelingen niet meegenomen)
Het gaat om de periode ná de laatste vragenlijst 4 maanden geleden

Bent u opgenomen geweest in een ziekenhuis? (Overnachting)
- ◯ Ja
- ◯ Nee

Wat was de reden dat u bent opgenomen? ______________________________

Wanneer bent u opgenomen geweest? ______________________________

Waar bent u opgenomen geweest?

- ◯ St. Antonius ziekenhuis
- ◯ Ander, verwijzend ziekenhuis
- ◯ Anders namelijk...

In welk ander ziekenhuis bent u opgenomen geweest? ______________________________

Op welke afdeling bent u opgenomen geweest?

- ◯ Cardiologie
- ◯ Anders namelijk...

Op welke andere afdeling bent u opgenomen? ______________________________

Bent u vaker opgenomen geweest?
- ◯ Ja
- ◯ Nee

Hoe veel keren bent u opgenomen (opname telt mee indien u tenminste 1 nacht in het ziekenhuis heeft doorgebracht)
- ◯ 2
- ◯ 3
- ◯ 4
- ◯ 5

Tweede opname

Wat was de reden dat u de tweede keer bent opgenomen? __________________________________

Wanneer bent u de tweede keer opgenomen geweest? __________________________________

Waar bent u de tweede keer opgenomen geweest?

◯ St. Antonius ziekenhuis
◯ Verwijzer: [baseline_arm_1][verwijzend_centrum]
◯ Anders namelijk...

In welk ander ziekenhuis bent u de tweede keer opgenomen geweest? __________________________________

Op welke afdeling bent u de tweede keer opgenomen geweest?

◯ Cardiologie
◯ Anders namelijk...

Op welke andere afdeling bent u de tweede keer opgenomen geweest? __________________________________

Derde opname

Wat was de reden dat u de derde keer bent opgenomen? ______________________

Wanneer bent u de derde keer opgenomen geweest? ______________________

Waar bent u de derde keer opgenomen geweest?

◯ St. Antonius ziekenhuis
◯ Verwijzer: [baseline_arm_1][verwijzend_centrum]
◯ Anders namelijk...

In welk ander ziekenhuis bent u de derde keer opgenomen geweest? ______________________

Op welke afdeling bent u de derde keer opgenomen geweest?

◯ Cardiologie
◯ Anders namelijk...

Op welke andere afdeling bent u de derde keer opgenomen? ______________________

Vierde opname

Wat was de reden dat u de vierde keer bent opgenomen? __________________________________

Wanneer bent u de vierde keer opgenomen geweest? __________________________________

Waar bent u de vierde keer opgenomen geweest?

◯ St. Antonius ziekenhuis
◯ Verwijzer: [baseline_arm_1][verwijzend_centrum]
◯ Anders namelijk...

In welk ander ziekenhuis bent u de vierde keer opgenomen geweest? __________________________________

Op welke afdeling bent u de vierde keer opgenomen geweest?

◯ Cardiologie
◯ Anders namelijk...

Op welke andere afdeling bent u de vierde keer opgenomen geweest? __________________________________

Vijfde opname

Wat was de reden dat u de vijfde keer bent opgenomen geweest?

____

Wanneer bent u de vijfde keer opgenomen geweest?

____

Waar bent u de vijfde keer opgenomen geweest?

◯ St. Antonius ziekenhuis
◯ Verwijzer: [baseline_arm_1][verwijzend_centrum]
◯ Anders namelijk...

In welk ander ziekenhuis bent u de vijfde keer opgenomen geweest?

____

Op welke afdeling bent u de vijfde keer opgenomen geweest?

◯ Cardiologie
◯ Anders namelijk...

Op welke andere afdeling bent u de vijfde keer opgenomen geweest?

____
